# Soil Bacterial Function Associated With Stylo (Legume) and Bahiagrass (Grass) Is Affected More Strongly by Soil Chemical Property Than by Bacterial Community Composition

**DOI:** 10.3389/fmicb.2019.00798

**Published:** 2019-04-12

**Authors:** Yang Zhou, Yongqiang Qin, Xiaodi Liu, Zengwei Feng, Honghui Zhu, Qing Yao

**Affiliations:** ^1^College of Horticulture, South China Agricultural University, Guangdong Province Key Laboratory of Microbial Signals and Disease Control, Guangdong Engineering Research Center for Litchi, Guangdong Engineering Research Center for Grass Science, Guangzhou, China; ^2^Guangdong Institute of Microbiology, State Key Laboratory of Applied Microbiology Southern China, Guangdong Provincial Key Laboratory of Microbial Culture Collection and Application, Guangzhou, China

**Keywords:** soil bacterial function, soil bacterial community composition, soil chemical property, soil enzyme activity, legume, grass

## Abstract

Soil microbes are driver of nutrient cycling, with microbial function affected by community composition and soil chemical property. Legume and grass are ubiquitous in many ecosystems, however, their differential effects on microbial function are less understood. Here we constructed compartmented rhizobox planted with stylo (*Stylosanthes guianensis*, legume) or bahiagrass (*Paspalum natatum*, grass) to compare their influences on bacterial function and to investigate the determinant of bacterial function. Soils in root compartment and in near (0–5 mm from root compartment) or far (10–15 mm from root compartment) rhizosphere were sampled. Soil chemical properties, bacterial community composition and function were characterized. Results indicate that plant species and distance significantly affected bacterial function. The activities of beta-xylosidase, nitrate reductase and phosphomonoesterase were higher in stylo soil than in bahiagrass soil, while leucine-aminopeptidase activity and *nosZ* abundance were *vice versa*. Rhizosphere effect was obvious for the activities of beta-glucosidase, beta-xylosidase, chitinase, and the abundances of AOB-*amoA*, *nirS*, *nosZ*. Statistical analysis revealed that soil chemical property was significantly associated with bacterial function, with a higher coefficient than bacterial community composition. These data suggest that stylo and bahiagrass differentially affect bacterial function, which is affected more strongly by soil chemical property than by community composition.

## Introduction

Rhizosphere is a critical micro-niche for soil microbes consuming rhizodeposits and in turn increasing the nutrient availability to themselves and plants (Knee et al., 2001; Paterson, 2003; Adesemoye et al., 2008). Accumulating evidence reveals the importance of rhizosphere microbes in plant growth performance (Adesemoye et al., 2008; Kolton et al., 2017). Previous studies clearly showed that soil microbial community structure was affected by the distance from rhizoplane (Marilley et al., 1998; Kandeler et al., 2002), and plants selected rhizosphere microbial community in terms of both structure and function (Kourtev et al., 2003; Kirk et al., 2005; Pivato et al., 2017). These reports highlight the necessity to reveal the influences of different plant species on rhizosphere microbial function in relation to community structure and composition.

Root deposits, especially root exudates, are the main compounds regulating microbial communities (Haichar et al., 2008; Guo et al., 2017). Difference in nutrient requirement of plants (plant nutritional strategy) can also result in the differential regulation of microbial community (Thion et al., 2016; Guyonnet et al., 2017). Legume and grass are two important plant groups with contrasting features regarding root exudates and nutrient requirements (Rovira, 1969; Isobe and Tsuboki, 1998; Gransee and Wittenmayer, 2000). For example, legume roots exuded more amino acids and sugar than grass roots, especially under low P level (Isobe and Tsuboki, 1998), and this difference in root exudate composition was confirmed by Gransee and Wittenmayer (2000). The difference in nutrient requirement between legume and grass was observed in intercropping system, where sorghum was more competitive for N and P than soybean (Ghosh et al., 2009), and barley took up more ^15^N than pea in both monocropping and intercropping system (Jensen, 1996). Therefore, it is expected that legume and grass differentially selected soil microbial community. Kirk et al. (2005) indicated that microbial community in the soil grown with perennial ryegrass (grass) was much different from that in the soil grown with alfalfa (legume) with a similarity of 60.9% as revealed by denaturing gradient gel electrophoresis. Zhou et al. (2017) demonstrated that effects of stylo (legume) and bahiagrass (grass) on microbial community structure and function were much different. The similarity of bacterial community between stylo soil and bulk soil was 79.4%, significantly lower than that (88.3%) between bahiagrass soil and bulk soil.

Soil microbial function is fundamental to terrestrial ecosystem services (Grigulis et al., 2013). Soil microbial function can be quantified with several techniques, among which soil enzyme assay and quantification of microbial functional gene abundance are frequently employed (Burns et al., 2013; Cui et al., 2015). Factors determining soil microbial function are diverse. In a laboratory experiment with arable field soil, Hünninghaus et al. (2017) indicated that changes in microbial function (decomposition of organic matter) could be related to changes in microbial community composition, which was supported by other studies (Ofek-Lalzar et al., 2014; Fernandez et al., 2016; Galand et al., 2018). This is because the members of a particular microbial taxon (for example, species or genus) are often associated with one or more common functions. However, soil chemical property is also important player in regulating soil microbial function. For example, pH strongly influenced bacterial *ureC* (encoding one of three structural subunits in the urease enzyme) abundance and urea hydrolysis (Fisher et al., 2017), and soil organic C, rather than soil P, greatly affected the *phoD* community structure and alkaline phosphatase activity in Karst soils (Hu et al., 2018). It seems that nutrients and substrates for microbial growth (e.g., organic C), or other abiotic factors (e.g., pH) changing the bioavailability of nutrients and substrates can regulate microbial functions. However, almost all of these studies explored only one side, either community composition or soil chemical property, and the comparison between the two sides is yet lacking. Consequently, further work is required to fill in the knowledge gap, revealing which is more important in regulating soil microbial function, community composition or soil chemical property.

Based on the aforementioned work and knowledge gap, we put forward the following hypotheses: (1) the influences of legume and grass on soil microbial community function are different, mainly due to their differential effects on soil chemical property and microbial community composition; and (2) soil chemical property is more effective than microbial community composition in affecting microbial function, because soil chemical property can affect microbial function both directly and indirectly via its influence on microbial community composition. In this study, two plant species (legume vs. grass) were grown in specially designed rhizoboxes to test these hypotheses. Soil bacterial community composition was characterized with 16S rRNA gene sequencing, and bacterial function was characterized using soil enzyme assay and bacterial functional gene abundance.

## Materials and Methods

### Soil and Plant Species

Soil used in this study was collected from a vegetable field (112.9045°E, 22.6720°N) in Heshan Hilly Interdisciplinary Experimental Station, Chinese Academy Science in Guangdong province, with laterite soil and a subtropical monsoon climate (Chen et al., 2012). Soil was air-dried, and then sieved through mesh of 2 mm pore size. The soil chemical properties were determined as following (g kg^−1^, average of three replicates): soil pH 6.05 (soil:ddH_2_O 1:2 v/w), total organic C 11.3, dissolved organic C 0.026, total N 0.84, total P 0.91, total K 10.10, available N 0.066, available P 0.184, available K 0.094. Total organic C and dissolved organic C were determined with titration after wet oxidation with H_2_SO_4_ and K_2_Cr_2_O_7_, CN, available P and available K were analyzed with alkali-hydrolyzed reduction diffusing method, colorimetric method and flame photometric method (Carter and Gregorich, 2008). Total N, total P and total K were measured using the Kjeldahl method, the molybdenum blue colorimetric method and the flame photometric method (Zhou et al., 2017). Stylo (*Stylosanthes guianensis*, legume) and bahiagrass (*Paspalum natatum*, grass) were selected in the this study because they have been grown widely as forage or cover crops in the tropical and subtropical areas (Cui et al., 2015).

### Rhizobox Construction, Plant Growth, and Harvest

The compartmented rhizobox (12 × 13.2 × 11.6 cm) was designed and constructed with acrylic plate comprising of one root compartment (3 cm) and two side compartments (4 cm). Root compartment was separated from side compartments with nylon mesh (25 μm pore size), preventing the root growth into side compartments. Each side compartment was further divided into several chambers with nylon mesh to establish the rhizosphere space with different distance from root compartment (Supplementary Figure S1). Soil was gently filled into the rhizobox so that the nylon mesh kept vertical and the soil surface in each compartment was the same in height. Totally, 1100 g soil was filled in each rhizobox.

Seeds of stylo and bahiagrass were purchased from market (Juemen Trading Co., Guangzhou), and germinated on filter paper after surface sterilization with 5% HClO_3_ for 15 min. After germination, 10 seedlings were transferred to root compartment and watered to the soil moisture of 18%. For each plant species, 5 rhizoboxes were prepared as 5 replicates. Plants were cultivated in a growth chamber with a light period of 12 h/12 h (200 μmol m^−2^ s^−1^) and a day/night temperature of 28°C/24°C, which was suitable for plant growth according to pilot trial. All rhizoboxes were watered every day to keep the soil moisture at 18% with weighing method. Harvest was performed after 3 months of plant growth. Shoots were cut and surface soil was discarded. All roots were taken out from soil, and any root debris was carefully picked out with forceps. To investigate the spatial variation of bacterial function, the soil in the root compartment, the near (0–5 mm from root compartment) and the far (10–15 mm from root compartment) rhizosphere were sampled (Supplementary Figure S1).

Soil samples were homogenized, sieved through a 2 mm mesh, and then divided into four aliquots. One aliquot (approximately 40 g) was air-dried for soil chemical property analysis, the second aliquot (approximately 20 g) was stored at 4°C for soil enzyme assay within 1 week, and the third aliquot (approximately 20 g) was stored at −80°C for the extraction of soil total DNA.

### Analysis of Soil Chemical Property

Soil chemical properties were determined according to a laboratory manual by Carter and Gregorich (2008). Soil moisture was determined by oven drying method. Total organic C and dissolved organic C were analyzed by titration after wet oxidation with H_2_SO_4_ and K_2_Cr_2_O_7_. Available N, available P and available K were quantified by alkali-hydrolyzed reduction diffusing method, colorimetric method and flame photometric method. Nitrate and ammonium were extracted with 2 M calcium chloride, and then determined with UV spectrophotometry and Nessler’s reagent colorimetry, respectively.

### Soil Enzyme Assay

To quantify soil microbial function, soil enzymes involved in the nutrient cycling were assayed, including urease, nitrate reductase, cellulase, alpha-glucosidase, cellobiosidase, beta-xylosidase, beta-glucosidase, chitinase, phosphomonoesterase, and leucine-aminopeptidase.

The activities of urease, nitrate reductase and cellulase were measured with spectrophotometric method. Urease assay was performed according to Tabatabai and Bremner (1972). Briefly, 0.25 g soil was slightly shaken in 125 μl of toluene for 15 min, and then urea dissolved in tris buffer (0.05 mol l^−1^, pH 9.0) with a concentration of 0.2 mol l^−1^ was added as substrate. The suspension was swirled for a few seconds and incubated at 37°C. After 24 h incubation, soil suspension was used to measure ammonium content by indophenols blue method on a microplate reader at 578 nm (Infinite M200, Tecan Co.). Nitrate reductase activity was determined by measuring the released nitrite incubating with diazo-coupling reagents (1% sulfanilamide in 3 N HCl and 0.01% N-(1-napthyl) ethylenediamine hydrochloride) (Lowe and Evans, 1964). Sodium nitrate (40 μmol l^−1^) dissolved in potassium succinate (40 μmol l^−1^, pH 6.8) was added to 0.1 g soil with a final volume of 0.6 ml. The reaction mixture was incubated for 1 h at 37°C, and stopped by 0.5 ml diazo-coupling reagents, coloration for 20 min, and was measured spectrophotometrically at 540 nm. Cellulase was assayed by measuring reducing power of resulting sugars with the aid of dinitrosalicylic acid (Miller et al., 1960). 0.25 g soil was added with Na-acetate buffer (50 mmol l^−1^, pH 6.0) containing 2% carboxymethylcellulose and 125 μl toluene, and then incubated for 1 h at 40°C. Then the mixture was heated in boiling water bath for 15 min. Reducing sugar in saccharification liquid was quantified with dinitrosalicylic acid method on a microplate reader at 550 nm.

Microplate fluorometric method was used to measure the activities of the remaining soil enzymes using fluorogenic substrates according to ISO/TS 22939 (2010). Briefly, soil suspensions containing 1 g fresh soil in 50 ml sodium acetate buffer (0.5 mol l^−1^, pH 5.5) were ultrasonically disaggregated and homogenized for 2 min with the output energy of 50 J s^−1^. One hundred and sixty microliter aliquots of each soil suspension were dispensed into 96-well black microplates (Jet Bio-Filtration Co.). Forty microliter l of substrate solution were added to each sample well with a final concentration of 500 μmol l^−1^ for alpha-glucosidase, cellobiosidase, beta-xylosidase and leucine-aminopeptidase, 550 μmol l^−1^ for beta-glucosidase and phosphomonoesterase, and 200 μmol l^−1^ for chitinase (Supplementary Table S1). Negative control (40 μl substrate solution and 160 μl sodium acetate buffer) and quench control (40 μl of 10 μmol l^−1^ 4-methylumbelliferone or 7-amino-4-methylcoumarin and 160 μl soil suspension) were also applied. Stock solution (500 μmol l^−1^) was used to obtain final concentration of 0, 1, 5, 10, 25, 50, 100, 200 μmol l^−1^ for 4-methylumbelliferone, and 0, 0.1, 0.5, 1, 5, 10, 25, 50 μM for 7-amino-4-methylcoumarin to generate standard curves. Plates were incubated for 3 h at 30°C shaking continuously. Fluorescence was measured using a microplate fluorometer (FLx800, BioTek) at 355 nm excitation and 460 nm emission.

### Soil DNA Extraction, MiSeq Sequencing, and Bioinformatic Analysis

Soil total DNA was extracted from 0.25 g soil using PowerSoil DNA Isolation Kit (Mobio Laboratories, Carlsbad, CA, United States) according to manufacturer’s protocol. DNA concentration and quality were monitored by NanoDrop 1000 spectrophotometer (Thermo Fisher Scientific, Waltham, MA, United States) and agarose gel electrophoresis.

Primer sets (Supplementary Table S2) with indexed adapters and barcode were used to amplify bacterial 16S rRNA V3-V4 genes (Fu et al., 2015). The amplicons were generated using the MetaVx^TM^ Library Preparation kit (GENEWIZ, Inc., South Plainfield, NJ, United States). PCR reactions were carried out in 25 μl with FastPfu DNA polymerase, and the amount of template DNA was 20 ng. Thermal cycling consisted of initial denaturation at 95°C for 3 min, followed by 24 cycles of denaturation at 94°C for 30 s, annealing at 57°C for 90 s, and elongation at 72°C for 60 s, finally 72°C for 5 min. DNA libraries were validated by Agilent 2100 Bioanalyzer (Agilent Technologies, Palo Alto, CA, United States), and quantified by Qubit 2.0 Fluorometer (Applied Biosystems, Carlsbad, CA, United States). Then the libraries were multiplexed and loaded on an Illumina MiSeq instrument according to manufacturer’s instructions (Illumina, San Diego, CA, United States). Sequencing was performed using a 300 bp paired-end (PE) configuration; image analysis and base calling were conducted with the MiSeq Control Software (MCS) embedded in the MiSeq instrument. The DNA sequences in this study have been deposited in NCBI Sequence Read Archive (SRA) with accession number SRP132275.

Raw sequence reads were de-multiplexed, quality-filtered, processed and analyzed using QIIME (Caporaso et al., 2012). Quality filtering on joined sequences was performed and sequence which did not fulfill the following criteria were discarded: sequence length < 200 bp, no ambiguous bases, mean quality score ≥ 20. Then the sequences were compared with the reference database (RDP Gold database) using UCHIME algorithm to detect chimeric sequence, and then the chimeric sequences and singletons were removed. Sequences were grouped into operational taxonomic units (OTUs) using the clustering program VSEARCH (1.9.6) against the Silva database pre-clustered at 97% sequence identity (Rognes et al., 2016). The representative sequence for each OTU was picked and used the RDP classifier to annotate taxonomic information. Samples were rarified to 34642 prior to downstream analyses.

To better understand the specific influence of plant species or distance from root compartment on bacterial community composition, we designated some OTUs as biomarkers of plant species or distance as described previously (Zhou et al., 2017). Biomarkers of each plant species referred to those OTUs with significantly higher abundance in one species than in the other species, and biomarkers of each distance referred to those OTUs with significantly higher abundance in one distance than in the other two distances.

### Quantification of Bacterial Functional Gene Abundance

Abundances of functional genes involved in nutrient cycling (Supplementary Table S1) were quantified using real-time quantitative PCR (RT-qPCR), including AOA-*amoA*, AOB-*amoA* (encoding ammonia monooxygenase of archaea and bacteria), *narG* (encoding nitrate reductase), *nirS* (encoding nitrite reductase), *nosZ* (encoding nitrous oxide reductase), *nifH* (encoding nitrogenase), *alp* (encoding phosphatase). The quantification of *nirK* was not conducted because their abundances were very low according to our pilot quantification after plant growth. The qPCR assay was conducted on a CFX96 Optical Real-Time Detection System (Bio-Rad Laboratories Inc., Hercules, CA, United States). The PCR primers and annealing temperatures are described in Supplementary Table S2. The reaction mixture contained 10 μl 2 × PCR buffer (iQ^TM^ SYBR^®^Green Supermix, Bio-Rad), 2.5 μl of each primer (2 μmol l^−1^), 1 μl of template DNA and then was added with sterile deionized water to 20 μl. The qPCR conditions were as follows: an initial denaturation at 95°C for 5 min; 40 cycles of denaturation at 95°C for 15 s, annealing at suitable temperature (Supplementary Table S2) for 30 s; melting curve analysis to confirm the specificity of amplification products. We normalized functional gene abundance with DNA yield of each sample, and the abundance was expressed as copy numbers per ng DNA. RT-qPCR was performed with three technical replicates and negative control for the extraction was included. Amplification efficiencies of 83–91% for functional genes were obtained. Agarose gel electrophoresis was performed to check the specificity of the amplification products.

### Data Analysis and Statistics

The differences in soil chemical properties and soil microbial functions (enzyme activity, functional gene abundance) among samples were estimated in R statistical software (version 3.1.0; R Development Core Team, 2012). Data normality and homoschedasticity were tested using Shapiro Wilk and Levene’s test, respectively, at *P* < 0.05. Data were log transformed if the variable did not meet the assumptions of parametric statistical tests. Pairwise comparisons between treatments were performed using Tukey’s HSD *post hoc* tests. The effect of the two fixed factors (plant species and distance) and their interaction on soil bacterial alpha diversity were also estimated in R. Beta diversity was calculated using weighted UniFrac distance and principal coordinate analysis (PCoA) performed. Permutational multivariate of variance (PERMANOVA) was further performed in PAST to access soil bacterial community structure based on soil bacterial community profiles (Hammer et al., 2001). Mantel test (999 permutations, Spearman’s rank correlation) was conducted in R to test the determinant of bacterial function.

To explore the direct and indirect relationships between soil chemical property, bacterial community composition and function, Partial Least Squares Path Modeling (PLS-PM) in the R “plspm” packages were used (Barberán et al., 2014). For PLS-PM, the measured soil chemical properties were used to perform Mantel test with Bray-Curtis dissimilarity of microbial function in the package “vegan.” Variables significantly related were retained for path analysis. PCoA was again performed to simplify the variance of community composition (Comp. 1 and Comp. 2) and function (Funct. 1 and Funct. 2), then the first and second coordinate were used to proxy the variance of microbial function across different samples (Wei et al., 2013).

## Results

### Soil Chemical Property as Affected by Plant Species and Distance

**FIGURE 1:**
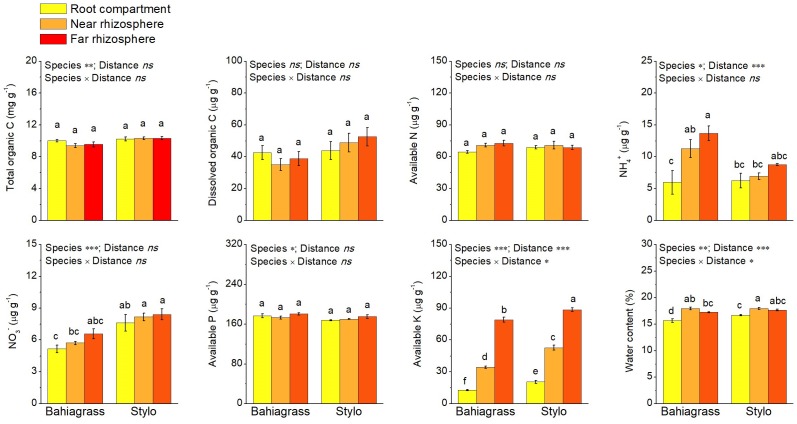
Soil chemical properties as affected by plant species and distance. Bars with different letters are significantly different (Tukey HSD test, *P* < 0.05). Near rhizosphere and far rhizosphere indicate the rhizosphere space of 0–5 mm and 10–15 mm apart from rhizoplane, respectively. Bahiagrass, *Paspalum natatum*; Stylo, *Stylosanthes guianensis*.

We found that plant species and/or distance affected most of the measured soil chemical properties after 3 months of growth, except that neither plant species nor distance affected available N (Supplementary Table S2). For plant species, NH_4_^+^ and available P in bahiagrass soil were significantly higher than those in stylo soil, while total organic C, dissolved organic C, NO_3_^−^, available K and water content in bahiagrass soil were significantly lower than those in stylo soil. For distance, rhizosphere effect was clear for NH_4_^+^, NO_3_^−^ and available K (Figure 1). It is worth noting that the rhizospere effect on NH_4_^+^ and NO_3_^−^ was stronger for bahiagrass than for stylo (Figure 1), demonstrating the difference in N requirement between legume and non-legume species. Moreover, available K exhibited a strong rhizosphere effect, with a sharp decrease from Rf to RC (Figure 1), indicating the large consumption by plants as well as the high mobility of K^+^.

### Soil Bacterial Community Structure and Composition as Affected by Plant Species and Distance

16S rRNA gene sequencing clearly revealed the influences of plant species and distance on the Simpson diversity of bacterial community (Table 1). However, Ace (abundance-based coverage estimator) and Chao1, measures of the predicted species richness in the environments, did not show significant difference between samples. Bacterial diversity in stylo soil was higher than that in bahiagrass soil; moreover, bacterial diversity increased along with the distance apart from roots, exhibiting obvious rhizosphere effect. There was not interaction between plant species and distance for all these parameters (Table 1).

**Table 1:** Soil bacterial community structure as affected by plant species and distance.

Plant species	Distance	Chao1	Simpson	Coverage
Bahiagrass	RC	1543.75 ± 19.86^a^	0.975 ± 0.006^b^	0.994 ± 0.000^a^
	Rn	1580.87 ± 29.73^a^	0.986 ± 0.003^ab^	0.995 ± 0.000^a^
	Rf	1539.75 ± 5.64^a^	0.990 ± 0.001^a^	0.995 ± 0.000^a^
Stylo	RC	1548.42 ± 12.14^a^	0.990 ± 0.000^a^	0.995 ± 0.000^a^
	Rn	1545.01 ± 5.45^a^	0.992 ± 0.000^a^	0.995 ± 0.000^a^
	Rf	1535.65 ± 15.17^a^	0.992 ± 0.000^a^	0.995 ± 0.000^a^
**TWO-WAY ANOVA (*P*-VALUE)**				
Plant species (S)		0.41	<0.01	0.06
Distance (D)		0.35	0.02	0.26
S × D		0.47	0.07	0.26

*Each value represents the average ± standard error. Different letters indicate significant difference in each column (Tukey HSD test, P = 0.05). Near rhizosphere (Rn) and far rhizosphere (Rf) indicate the rhizosphere space of 0–5 mm and 10–15 mm apart from rhizoplane, respectively. RC, root compartment; Bahiagrass, Paspalum natatum; Stylo, Stylosanthes guianensis.*

The main phyla (relative abundance > 5%) in rhizosphere were Proteobacteria, Acidobacteria, Actinobacteria, Firmicutes, Chloroflexi, Bacteroidetes, and both plant species and distance from root compartment affected their abundance (Supplementary Figure S2). Bahiagrass enriched significantly higher abundance of Proteobacteria (*P* = 0.004) than stylo, while stylo enriched significantly higher abundance of Actinobacteria (*P* = 0.031) than bahiagrass. Acidobacteria abundance (*P* = 0.004) and Bacteroidetes abundance (*P* = 0.012) significantly rose with increased distance from root compartment, while Actinobacteria abundance (*P* = 0.006) significantly decreased with increased distance (Supplementary Figure S2).

**FIGURE 2:**
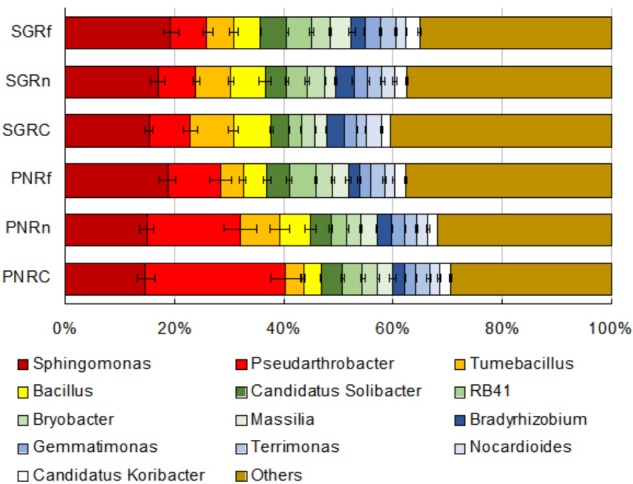
Abundances of soil bacterial taxa (genera with abundance > 1%) as affected by plant species and distance. PN, bahiagrass (*Paspalum natatum*); SG, stylo (*Stylosanthes guianensis*). RC, Rn, and Rf indicate root compartment, the near (0–5 mm) and the far (10–15 mm) rhizosphere.

The main bacterial genera (abundance > 1%) in soil were shown in Figure 2. The abundance of *Pseudarthrobacter* was much higher in bahiagrass soil than that in stylo soil, while the abundance of *Tumebacillus* and *Bacillus* was much lower in bahiagrass soil. We identified some biomarker bacteria of both plant species and of the soil with different distance from root compartment (Supplementary Dataset). The biomarker bacteria of bahiagrass were *Pseudarthrobacter*, *Sphingomonas*, Candidatus *Solibacter*, and some uncultured taxa, in contrast, the biomarker bacteria of stylo were *Tumebacillus*, *Bacillus drentensis*, *Bradyrhizobium*, *Streptomyces*, and some uncultured taxa (Table 2). For bahiagrass, the biomarker bacteria of root compartment were *Bromus tectorum*, *Oryza meyeriana*, and *Luteolibacter*; those of near rhizosphere were *Variibacter*, Rhodospirillales I-10; and those of far rhizosphere were Candidatus *Koribacter*. For stylo, the biomarker bacteria of root compartment were *Streptomyces*, *Mesorhizobium*, and Chitinophagaceae; those of far rhizosphere were uncultured Nitrosomonadaceae, Holophagae Subgroup 7, and some unknown taxa (Table 3). The difference in biomarker bacterial taxa highlights the influences of plant species and distance on soil bacterial community.

**Table 2:** Biomarkers of different plant species as revealed by soil bacteria (OTUs) enriched in root compartment.

OTU code	Abundance		Biomarker	Taxon
	Bahiagrass	Stylo		
OTU1	4779 ± 676^a^	1373 ± 216^b^	Bahiagrass	*Pseudarthrobacter*
OTU38	236 ± 13^a^	178 ± 6^b^	Bahiagrass	*Sphingomonas*
OTU158	225 ± 12^a^	171 ± 9^b^	Bahiagrass	Candidatus *Solibacter*
OTU27	220 ± 6^a^	148 ± 1b	Bahiagrass	Uncultured Holophagae
OTU81	192 ± 10^a^	107 ± 6^b^	Bahiagrass	Uncultured Acidobacteriaceae
OTU10	407 ± 59^b^	925 ± 134^a^	Stylo	*Tumebacillus*
OTU18	323 ± 23^b^	751 ± 41^a^	Stylo	*Bacillus drentensis*
OTU3	420 ± 22^b^	558 ± 16^a^	Stylo	Uncultured Rhodobiaceae
OTU12	371 ± 28^b^	498 ± 9^a^	Stylo	*Bradyrhizobium*
OTU237	61 ± 9^b^	224 ± 33^a^	Stylo	*Streptomyces*

*For each plant species, only the top 5 OTUs (abundance > 0.1%) with significant difference in abundance between plant species were listed. Bahiagrass, Paspalum natatum; Stylo, Stylosanthes guianensis.*

**Table 3:** Biomarkers of different distance in the rhizosphere of two plant species as revealed by soil bacteria (OTUs) enriched.

Plant species	OTU code	Abundance			Biomarker	Taxon
		RC	Rn	Rf		
Bahiagrass	OTU1524	199 ± 70^a^	1 ± 1b	0^b^	RC	*Bromus tectorum*
	OTU92	160 ± 58^a^	0^b^	0^b^	RC	*Oryza meyeriana*
	OTU617	84 ± 20^a^	16 ± 4^b^	1 ± 0^b^	RC	*Luteolibacter*
	OTU180	110 ± 6^b^	142 ± 7^a^	107 ± 4^b^	Rn	*Variibacter*
	OTU19	54 ± 7^b^	87 ± 3^a^	48 ± 3^b^	Rn	Rhodospirillales I-10
	OTU559	33 ± 6^b^	32 ± 6^b^	63 ± 3^a^	Rf	Candidatus *Koribacter*
Stylo	OTU237	224 ± 33^a^	67 ± 18^b^	31 ± 3^b^	RC	*Streptomyces*
	OTU57	151 ± 14^a^	65 ± 11^b^	32 ± 3^b^	RC	*Mesorhizobium*
	OTU299	99 ± 6^a^	66 ± 1^b^	78 ± 4^b^	RC	Chitinophagaceae
	OTU39	204 ± 10^c^	272 ± 7^b^	318 ± 10^a^	Rf	Uncultured Nitrosomonadaceae
	OTU35	94 ± 6^b^	127 ± 13^b^	173 ± 3^a^	Rf	Holophagae Subgroup 7
	OTU20	35 ± 16^b^	107 ± 11^b^	239 ± 23^a^	Rf	Bacteria

*Only the top 3 OTUs (abundance > 0.1%) with significant difference in abundance among three distances were listed. No biomarker was observed for near rhizosphere of stylo plants. Bahiagrass, Paspalum natatum; Stylo, Stylosanthes guianensis. RC, root compatment; Rn, the near rhizosphere (0–5 mm); Rf, the far rhizosphere (10–15 mm).*

**FIGURE 3:**
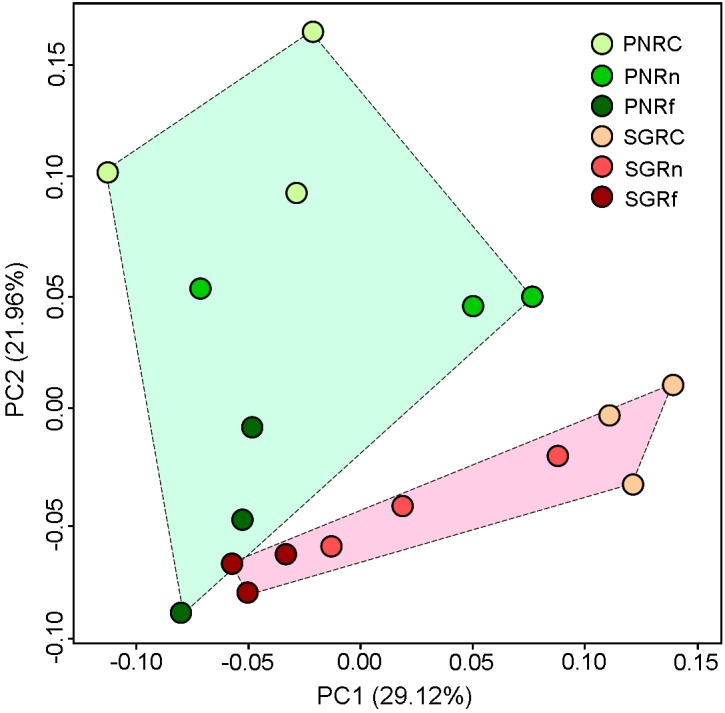
Principal coordinates analysis of soil bacterial community (OUT abundance) as revealed by weighted UniFrac dispersion. PN, bahiagrass (*Paspalum natatum*); SG, stylo (*Stylosanthes guianensis*). RC, Rn, and Rf indicate root compartment, the near (0–5 mm) and the far (10–15 mm) rhizosphere.

Permutational multivariate analysis of variance (PERMANOVA) based on 16S rRNA gene sequencing indicates that both plant species (*P* < 0.001) and distance (*P* < 0.001) significantly affected bacterial community structure with significant interaction between them (*P* < 0.01) (Table 4). PCoA also suggests the distinct bacterial community structures between plant species, and among the different distance (Figure 3). Moreover, the dissimilarity in bacterial community structure between plant species decreased along with the distance apart from roots.

**Table 4:** Permutational multivariate analysis of variance (PERMANOVA) results of soil bacterial community based on 16S rRNA gene sequencing, influences of plant species and distance.

Source	*SS*	*DF*	*MS*	*F*	*P*
Plant species	0.063	1	0.063	4.530	<0.001
Distance	0.096	2	0.048	3.479	<0.001
Interaction	0.061	2	0.031	2.218	<0.01
Residual	0.166	12	0.014		

### Soil Enzyme Activity as Affected by Plant Species and Distance

**FIGURE 4:**
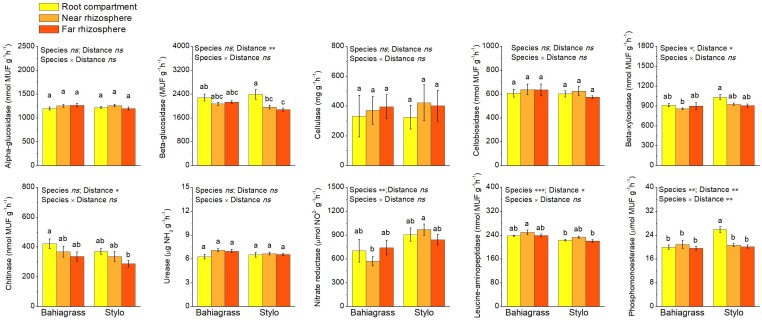
Soil enzyme activities as affected by plant species and distance. Bars with different letters are significantly different (Tukey HSD test, *P* < 0.05). Near rhizosphere and far rhizosphere indicate the rhizosphere space of 0–5 mm and 10–15 mm apart from rhizoplane, respectively. Bahiagrass, *Paspalum natatum*; Stylo, *Stylosanthes guianensis*.

The influences of plant species and distance on soil enzyme activity were shown in Figure 4. Plant species and/or distance significantly affected the activities of soil enzymes except alpha-glucosidase, cellulase, cellobiosidase and urease (Figure 4 and Supplementary Table S3). Leucine-aminopeptidase activity was higher but the activities of beta-xylosidase, nitrate reductase and phosphomonoesterase were lower in bahiagrass soil than in stylo soil (Figure 4 and Supplementary Table S3). On the other hand, the activities of beta-glucosidase, beta-xylosidase and chitinase decreased along with the distance apart from roots, indicating the rhizosphere effect. However, the rhizosphere effect on phosphomonoesterase was observed only in stylo soil but not in bahiagrass soil (Figure 4 and Supplementary Table S3).

### The Abundances of Functional Genes as Affected by Plant Species and Distance

**FIGURE 5:**
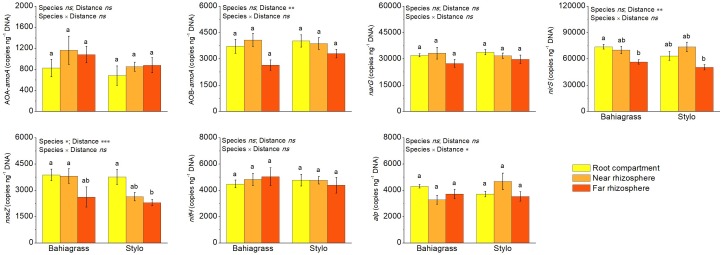
The abundances of bacterial functional genes as affected by plant species and distance. Bars with different letters are significantly different (Tukey HSD test, *P* < 0.05). Near rhizosphere and far rhizosphere indicate the rhizosphere space of 0–5 mm and 10–15 mm apart from rhizoplane, respectively. Bahiagrass, *Paspalum natatum*; Stylo, *Stylosanthes guianensis*.

Apart from soil enzyme activity, bacterial functional gene abundance was also quantified to represent soil bacterial function. RT-qPCR reveals that the functional genes were significantly affected by plant species and/or distance except AOA-*amoA*, *narG*, *nifH*, and *alp* (Figure 5). Plant species significantly affected only *nosZ*, with *nosZ* abundance higher in bahiagrass soil than stylo soil (Figure 5 and Supplementary Table S3). On the other hand, distance significantly affected AOBA-*amoA*, *nirS*, and *nosZ*, with their abundances decreasing along with the distance apart from roots (Figure 5 and Supplementary Table S3). For *alp*, there was an interaction between plant species and distance.

### Determinant of Soil Bacterial Function

**FIGURE 6:**
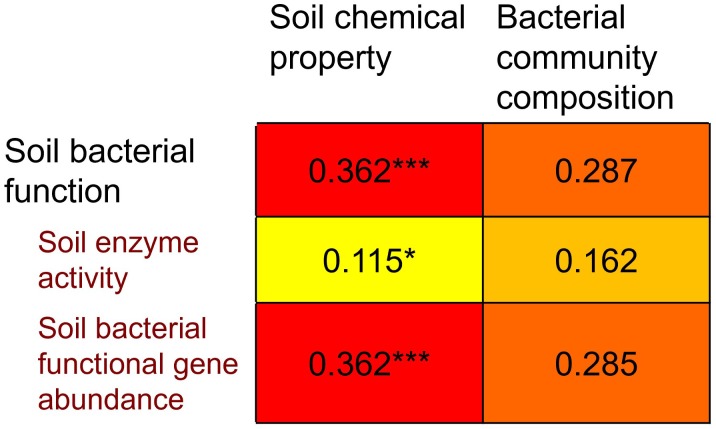
Spearman’s rank correlations (*r*-values), determined via Mantel test, relating to Bray-Cutris distance dissimilarity matrices generated from soil bacterial community composition at genus level, and to Euclidean distance dissimilarity matrices generated from soil chemical property and soil microbial function. Soil microbial function is further divided into two categories: soil enzyme activity and soil bacterial functional gene abundance.

Mantel test indicates that soil bacterial function was significantly correlated with soil chemical property (*P* = 0.002) but not with bacterial community composition (*P* = 0.997) (Figure 6), suggesting that bacterial function is affected more strongly by soil chemical property than by bacterial community composition. Furthermore, we divided bacterial function into two categories, e.g., soil enzyme activity and bacterial functional gene abundance. Soil enzyme activity (*P* = 0.048) and bacterial functional gene abundance (*P* = 0.017) were significantly correlated with soil chemical property, respectively, while they were not significantly correlated with bacterial community composition (*P* = 0.100 and 0.996, respectively) (Figure 6).

**FIGURE 7:**
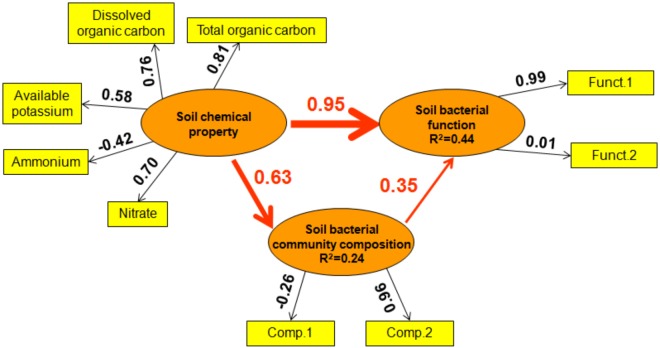
Directed graph of the Partial Least Squares Path Model (PLS-PM). Observed (i.e., measured) variables are represented in a rectangular form, while latent variables (i.e., constructs) are represented in an elliptical form. The loadings (the correlations between a latent variable and its observed variables) are indicated as the values in rectangles, and the path coefficients (between latent variables) and the coefficients of determination (*R*^2^ in ellipses) are calculated after 999 bootstraps. The Model was assessed using the Goodness of Fit (GoF) statistic, a measure of the overall prediction performance. The GoF was 0.42.

Furthermore, PLS-PM was conducted to illustrate the direct and indirect relationships of soil bacterial function and soil chemical property, bacterial community composition. In general, soil chemical property produced a larger direct effect on soil bacterial function than bacterial community composition (effect size 0.95 vs. 0.35, Figure 7). Additionally, soil chemical property produced an indirect effect on soil bacterial function *via* its influence on bacterial community composition (effect size 0.63, Figure 7).

## Discussion

In this study, we measured bacterial functional gene abundance and soil enzyme activities (products of functional genes) to indicate bacterial functions. The primers used in this study for bacterial functional genes were bacteria-specific, therefore the functional gene abundance could be well indicative of bacterial functions. However, in contrast, soil enzymes could not be exclusively indicative of bacterial functions because soil enzymes can be originated not only from bacteria but also from other organisms, such as fungi and plants. In this context, it is not surprising that the bacterial community composition was more closely related (higher correlation coefficient) with functional gene abundance than with soil enzyme activity. Although functional genes are inherent to bacterial taxa, their transcription and translation are greatly regulated by environmental conditions (Schreiber et al., 2007; Kim et al., 2016; Hockenberry et al., 2017). Taking nitrate respiration as example, a serial of functional genes (*nar*, *nir*) are regulated by anaerobic condition and nitrate presence *via* transcription network in *Pseudomonas aeruginosa* (Schreiber et al., 2007). The Shine-Dalgarno (SD) sequence motif is the dominant mechanism of translation initiation in bacteria, and Hockenberry et al. (2017) illustrated that environmental factor (temperature) influenced the translation *via* SD sequence mechanism by comparing mesophiles and thermophiles. Therefore, it is reasonable to find that functional gene abundance was significantly correlated with soil chemical property in this study. Additionally, soil chemical property can exert direct influences on soil enzyme activity. A meta-analysis pooling data from 40 ecosystems indicated that pH affected β-1,4-glucosidase, cellobiohydrolase, β-1,4-N-acetylglucosaminidase, phosphatase, leucine aminopeptidase, phenol oxidase and peroxidase activities, and soil organic matter affected the former four enzyme activities (Sinsabaugh et al., 2008). As aforementioned, however, it is likely that soil chemical property correlate with functional gene abundance more closely than with soil enzyme activity, as revealed in this study.

Our results did not support a significant correlation between community composition and function. The factor weakening the correlation between community composition and functions can be the bacterial multifunctionality and functional redundancy. There are some versatile bacterial taxa with multiple functions in diverse ecosystems (Hartmann et al., 2015), e.g., some isolates from *Bacillus* and *Pseudomonas* (Jeong et al., 2010; Chaturvedi et al., 2014). Ryan et al. (2009) suggested that the functioning of versatile isolates depends on environmental conditions. This highlights the critical role of soil chemical property in shaping bacterial community function as revealed in this study. Functional redundancy is normally at high level in microbes (Nannipieri et al., 2017). Banerjee et al. (2016) showed the microbial functional redundancy in an organic matter decomposition experiment, where the abundance of dominant decomposer changed 4–300-fold with the decomposition rate maintaining similar. In this context, interpreting bacterial function with taxonomic information can be difficult, although 16S rRNA marker gene can be sequenced to predict microbial functions using a computational approach (PICRUSt) (Langille et al., 2013) or other tools. In general, for the bacterial community functions, soil chemical properties and bacterial community composition can be regarded as external and internal affecting factors, respectively. In this study, it seems that external factors are more influential than internal factor to shape the bacterial function profile.

Plants can alter both bacterial community composition and function *via* the modification of soil chemical property by roots. For example, Wang et al. (2015) suggested the importance of soil physiochemical properties in determining the activities of ammonia oxidizers and nitrite oxidizers. In this study, we found that the soil chemical property was much different between bahiagrass and stylo, which might be resulted from the difference in root uptake and root exudation and could contribute greatly to the difference in bacterial community composition and function.

It is well known that rhizosphere effect depends on the distance from rhizoplane (Wang et al., 2014; Razavi et al., 2016). In this study, we set up the near and far rhizosphere using compartmentation with nylon mesh to characterize the rhizosphere effect. We found that soil bacterial function showed strong rhizosphere effect, in terms of C cycling (activities of beta-glucosidase and beta-xylosidase), N cycling (chitinase activity, abundances of *narG*, *nirS* and *nosZ*), and P cycling (phosphomonoesterase activity, *alp* abundance). It seems that the rhizosphere effect on N cycling is more extensive, probably due to the complexity of N cycling in soil. The differential influences of stylo and bahiagrass on soil chemical properties were demonstrated in this study and previously (Zhou et al., 2017), which might further lead to different bacterial community composition (or specific populations) and functions, as indicated in this study. The specifically recruited microbial assembly and the associated functions can support better plant growth performance, especially under environmental stresses (Bai et al., 2015), depending on plant species.

## Conclusion

In conclusion, with stylo (legume) and bahiagras (grass) for comparison, we found that they differentially affected soil chemical property. The contents of organic C, NO_3_^−^ and available K were higher while those of NH_4_^+^ and available P were lower in stylo soil. The biomarker bacteria of stylo and bahiagrass were *Tumebacillus*, *Bacillus drentensis*, *Bradyrhizobium*, *Streptomyces*, and *Pseudarthrobacter*, *Sphingomonas*, Candidatus *Solibacter*, respectively. Soil bacterial function was characterized by soil enzyme activity and bacterial functional gene abundance. The activities of beta-xylosidase, nitrate reductase and phosphomonoesterase in stylo soil were higher than those in bahiagrass soil, while leucine-aminopeptidase activity and *nosZ* abundance in stylo soil were lower than those in bahiagrass soil. Spearman’s correlation determined by Mantel test suggests a significant relationship between soil chemical property and bacterial function. Path analysis indicated a stronger direct and indirect effect of soil chemical property on bacterial function than that of bacterial community composition. Additionally, rhizosphere effect was observed for some parameters of soil chemical property, bacterial community composition, and bacterial function, especially those involved in N cycling. In conclusion, these data suggest that bacterial function is affected more strongly by soil chemical property than by bacterial community composition, and legume and grass can affect soil bacterial function in different patterns.

## Author Contributions

QY, HZ, and YZ designed this study. YZ and YQ performed the experiments. ZF and XL contributed to the molecular analysis. YZ and QY analyzed the data. YZ, QY, and HZ wrote the manuscript with help from other co-authors.

## Conflict of Interest Statement

The authors declare that the research was conducted in the absence of any commercial or financial relationships that could be construed as a potential conflict of interest.
