# Supplementary material for: Soil Bacterial Function Associated With Stylo (Legume) and Bahiagrass (Grass) Is Affected More Strongly by Soil Chemical Property Than by Bacterial Community Composition
Source: Front Microbiol. 2019 Apr 12;10:798. doi: 10.3389/fmicb.2019.00798 (PMC6473644; doi:10.3389/fmicb.2019.00798)
Supplement: Supplementary file 1 [file Data_Sheet_1.doc]

**Figure S1.** Photographs demonstrating the construction of rhizobox and sampling areas of RC (root compartment), Rn (near rhizosphere), Rf (far rhizosphere). Each rhizobox was divided into several space with nylon mesh (25 μm pore size) for convenient sampling. A and C are bahiagrass seedlings (*Paspalum natatum*, PN); B and D are common stylo seedlings (*Stylosanthes guianensis*, SG). The rhizoboxes were constructed with opaque black acrylic plate (5 mm thick).

**
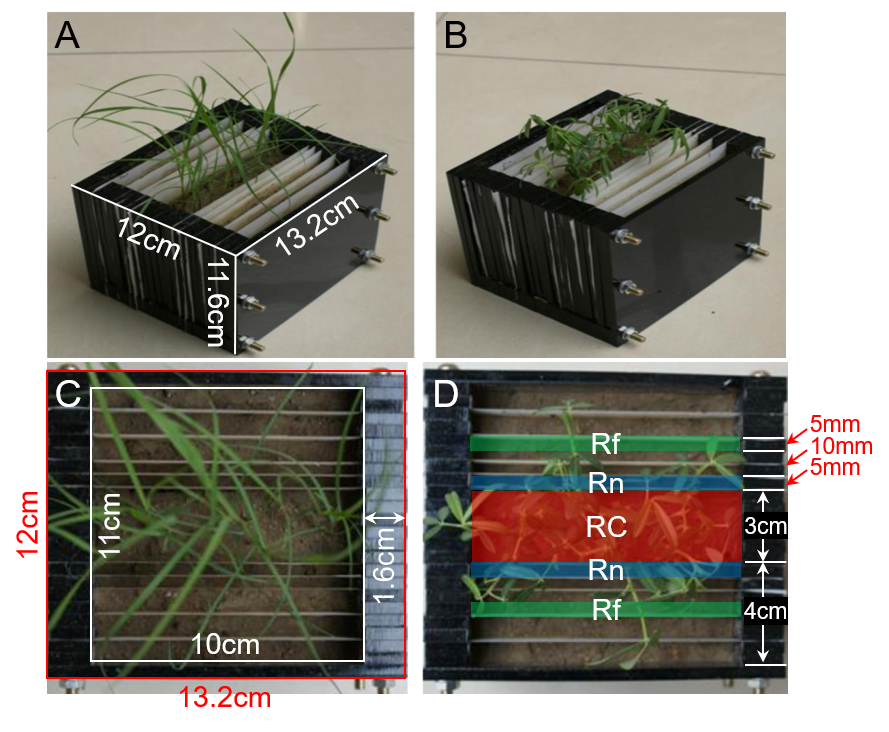
**

**Figure S2.** The relative abundance of bacterial taxa at phylum level as affected by plant species and distance from root compartment. A, Combined effects of plant species and distance; B, Effects of distance; C, Effects of plant species. PN, bahiagrass; SG, stylo; RC, root compartment; Rn, near rhizosphere; Rf, far rhizosphere.


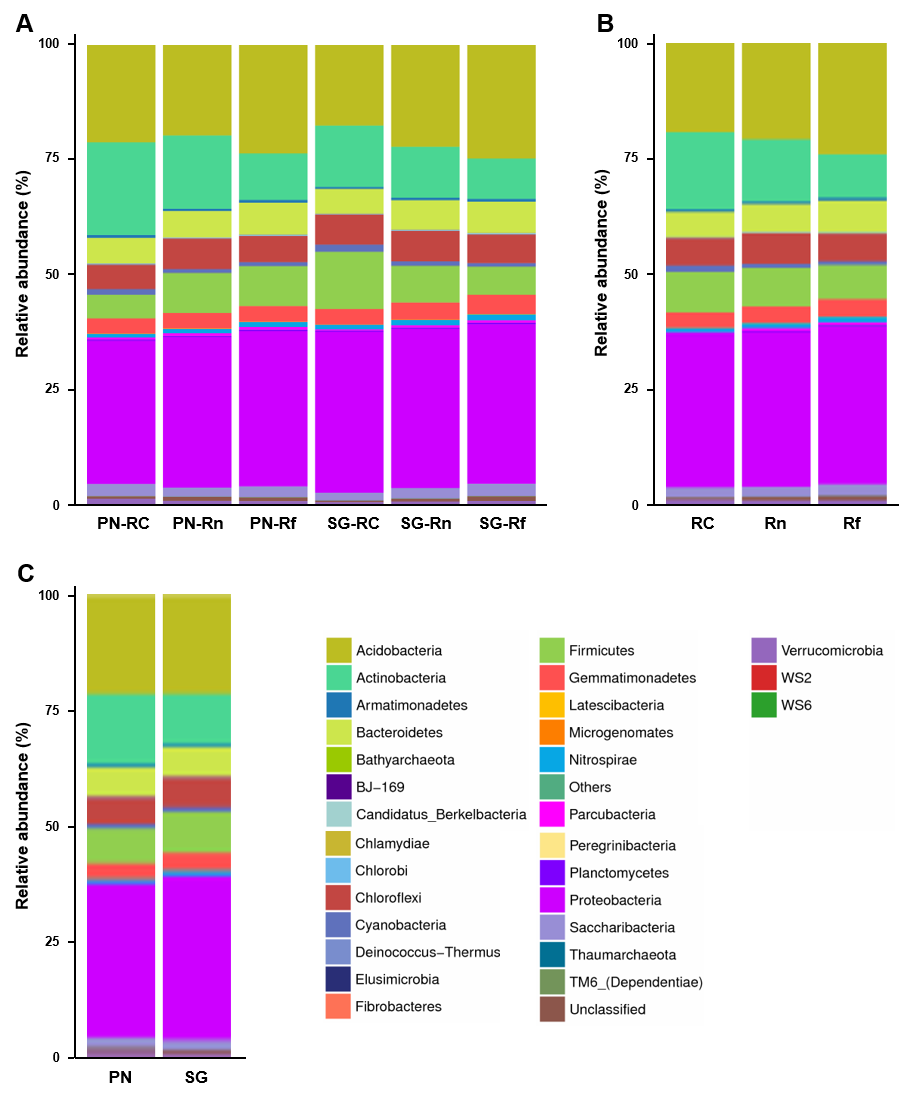


**Table S1.** Commercially available fluorogenic substrates for the enzyme assay with microplate fluorometric method in this study and their respective final concentrations in reaction system. MUF, methylumbelliferyl; AMC, 7-amino-4-methylcoumarin.

| Enzymes | Fluorogenic substrates | Final substrate concentration |
| --- | --- | --- |
| alpha-glucosidase | 4-MUF-alpha-D-glucopyranoside | 500 μmol l^-1^ |
| cellobiosidase | 4-MUF-beta-cellobiopyranoside | 500 μmol l^-1^ |
| beta-xylosidase | 4-MUF-beta-D-xylopyranoside | 500 μmol l^-1^ |
| beta-glucosidase | 4-MUF-beta-D-glucopyranoside | 550 μmol l^-1^ |
| chitinase | 4-MUF-N-acetyl-beta-D-glucosaminide | 200 μmol l^-1^ |
| phosphomonoesterase | 4-MUF-phosphate | 550 μmol l^-1^ |
| leucine-aminopeptidase | L-leucine-AMC | 500 μmol l^-1^ |

**Table S2.** PCR primers and annealing temperatures used in this study, and the corresponding references.

**G**

| Functional genes | Primers | Primer sequences (5’ –3’) | Annealing temperature | References |
| --- | --- | --- | --- | --- |
| V3-V4 | Forward | CCTACGGRRBGCASCAGKVRVGAAT | 57 ^o^C | Fu et al., 2015 |
|  | Reverse | GGACTACNVGGGTWTCTAATCC |  |  |
| AOA-*amoA* | amoAF | STAATGGTCTGGCTTAGACG | 55 ^o^C | Wang et al., 2015 |
|  | amoAR | GCGGCCATCCATCTGTATGT |  |  |
| AOB-*amoA* | amoA1F | GGGGTTTCTACTGGTGGT | 55 ^o^C | Wang et al., 2015 |
|  | amoA2R | CCCCTCTGCAAAGCCTTCTTC |  |  |
| *narG* | narG-F | TCGCCSATYCCGGCSATGTC | 58 ^o^C | Bru et al., 2007 |
|  | narG-R | GAGTTGTACCAGTCRGCSGAYTCSG |  |  |
| *nirS* | cd3aF | GTSAACGTSAAGGARACSGG | 57 ^o^C | Geets et al., 2007 |
|  | R3cd | GASTTCGGRTGSGTCTTGA |  |  |
| *nosZ* | nosZ2F | CGCRACGGCAASAAGGTSMSSGT | 60 ^o^C | Henry et al., 2006 |
|  | nosZ2R | CAKRTGCAKSGCRTGGCAGAA |  |  |
| *nifH* | PolF | TGCGAYCCSAARGCBGACTC | 58 ^o^C | Orr et al., 2012 |
|  | PolR | ATSGCCATCATYTCRCCGGA |  |  |
| *alp* | F730 | CAGTGGGACGACCACGAGGT | 57 ^o^C | Sakurai et al., 2008 |
|  | R1101 | GAGGCCGATCGGCATGTCG |  |  |

**References**

Bru D, Sarr A, Philippot L. Relative abundances of proteobacterial membrane-bound and periplasmic nitrate reductases in selected environments. *Appl Environ Microbiol* 2007;**73**:5971–5974.

Fu SF, He S, Shi XS *et al*. The chemical properties and microbial community characterization of the thermophilic microaerobic pretreatment process. *Bioresource Tech* 2015;**198**:497–502.

Geets J, Cooman M, Wittebolle L *et al*. Real-time PCR assay for the simultaneous quantification of nitrifying and denitrifying bacteria in activated sludge. *Appl Microbiol Biotechnol* 2007;**75**:211–221.

Henry S, Bru D, Stres B *et al*. Quantitative detection of the *nosZ* gene, encoding nitrous oxide reductase, and comparison of the abundances of 16S rRNA, *narG*, *nirK* and *nosZ* genes in soils. *Appl Environ Microbiol* 2006;**72**:5181–5189.

Orr CH, Leifert C, Cummings SP *et al*. Impacts of organic and conventional crop management on diversity and activity of free-living nitrogen fixing bacteria and total bacteria are subsidiary to temporal effects. *PLoS ONE* 2012;**7**:e52891.

Sakurai M, Wasaki J, Tomizawa Y *et al*. Analysis of bacterial communities on alkaline phosphatase genes in soil supplied with organic matter. *Soil Sci Plant Nutr* 2008;**54**:62–71.

Wang B, Zhao J, Guo Z *et al*. Differential contributions of ammonia oxidizers and nitrite oxidizers to nitrification in four paddy soils. *ISME J* 2015;**9**:1062–1075.

**Table S3.** The ANOVA summarizing the influences (*P* values) of plant species and distance on each parameter of soil chemical property, soil enzyme activity, and bacterial functional gene abundance. Each parameter was subjected to log transformation (non-normal distribution) or not (normal distribution) before ANOVA.

| Category | Parameters | Plant species (P) | Distance (D) | P × D |
| --- | --- | --- | --- | --- |
| Soil chemical property | total organic C | 0.002 | 0.466 | 0.282 |
|  | dissolved organic C | 0.025 | 0.726 | 0.301 |
|  | available N | 0.988 | 0.108 | 0.158 |
|  | NH_4_^+^ | 0.037 | 0.001 | 0.063 |
|  | NO_3_^-^ | 0.000 | 0.088 | 0.760 |
|  | available P | 0.035 | 0.181 | 0.443 |
|  | available K | 0.000 | 0.000 | 0.011 |
|  | water content | 0.002 | 0.000 | 0.046 |
| Soil enzyme activity | alpha-glucosidase | 0.635 | 0.269 | 0.286 |
|  | beta-glucosidase | 0.150 | 0.002 | 0.153 |
|  | cellulase | 0.771 | 0.564 | 0.913 |
|  | cellobiosidase | 0.464 | 0.713 | 0.697 |
|  | beta-xylosidase | 0.014 | 0.047 | 0.267 |
|  | chitinase | 0.087 | 0.034 | 0.948 |
|  | urease | 0.277 | 0.088 | 0.210 |
|  | nitrate reductase | 0.004 | 0.929 | 0.263 |
|  | leucine-aminopeptidase | 0.000 | 0.032 | 0.909 |
|  | phosphomonoesterase | 0.006 | 0.004 | 0.002 |
| Bacterial functional gene abundance | AOA-*amoA* | 0.110 | 0.203 | 0.853 |
|  | AOB-*amoA* | 0.338 | 0.010 | 0.427 |
|  | *narG* | 0.607 | 0.100 | 0.628 |
|  | *nirS* | 0.943 | 0.006 | 0.232 |
|  | *nosZ* | 0.026 | 0.001 | 0.262 |
|  | *nifH* | 0.734 | 0.941 | 0.627 |
|  | *alp* | 0.570 | 0.488 | 0.036 |
